# Reproducibility of science: Fraud, impact factors and carelessness

**DOI:** 10.1016/j.yjmcc.2017.10.009

**Published:** 2018-01

**Authors:** D.A. Eisner

**Affiliations:** Unit of Cardiac Physiology, Division of Cardiovascular Sciences, Manchester Academic Health Sciences Centre, 3.18 Core Technology Facility, University of Manchester, 46 Grafton ST, Manchester M13 9NT, UK

**Keywords:** Fraud, Reproducibility

## Abstract

There is great concern that results published in a large fraction of biomedical papers may not be reproducible. This article reviews the evidence for this and considers some of the factors that are responsible and how the problem may be solved. One issue is scientific fraud. This, in turn, may result from pressures put on scientists to succeed including the need to publish in “high impact” journals. I emphasise the importance of judging the quality of the science itself as opposed to using surrogate metrics. The other factors discussed include problems of experimental design and statistical analysis of the work. It is important that these issues are addressed by the scientific community before others impose draconian regulations.

## Highlights

• Much of science cannot be reproduced; this article reviews the causes.
• Fraud, sometimes encouraged by the need to publish in high impact journals contributes.
• Another, major factor, is poor experimental design and statistical analysis.
• The scientific community needs to reform before change is thrust upon it.

## 1 Introduction

Science progresses by findings from one researcher or group being advanced by others. This is often summed up as, “*If I have seen further, it is by standing on the shoulders of giants*” (This quotation is widely attributed to Isaac Newton but may originally come from the 12th Century French philosopher, Bernard of Chartres [1].) In contrast, anyone who has read commentary articles in scientific journals or scanned the lay press will be aware of the current interest in the idea that we live in a dystopia where much of the research carried out in basic, preclinical biomedical science may not be reproducible. A major writer in this field John Ioannidis [2] has written a series of provocative articles including one with the title “*Why most research findings are false*”. The pharmaceutical industry is also very concerned. Scientists at Bayer became worried by problems in drug development while using results of preclinical studies carried out outside of the company. They were only able to replicate the underpinning research, in about one quarter of studies [3]. Similarly, Amgen scientists could only confirm findings in 11% of “landmark” studies [4]. The issue also worries funders. Francis Collins (NIH Director) states that “*A growing chorus of concern, from scientists and laypeople, contends that the complex system for ensuring the reproducibility of biomedical research is failing and is in need of restructuring*” [5]. In other words, despite the peer review of both the grants that fund the work and the papers in which it is published, serious errors are being published.

If a significant amount of science is, indeed, not reproducible then the consequences are potentially grim for us all. On the one hand, researchers will waste their time and money basing their research on the erroneous publications of others. On the other, it may make the public and politicians less ready to support and fund research. Furthermore, as discussed later, some of the remedies proposed to improve reproducibility will be cumbersome and may hamper scientific activity. In this article I will therefore address two questions. (1) What are the causes of the lack of reproducibility? (2) How might the problem be solved? As regards to the first question, many factors could be responsible. The causes can broadly be divided into two categories: (i) fraud and (ii) poor experimental design, execution and analysis.

## 2 Fraud

There are some very famous cases of fraud which range from inappropriate deletion of “outliers” to data manipulation to pure invention. Perhaps the Piltdown man is one of the first famous frauds of the modern scientific era. In 1912 this was claimed to be the evolutionary “missing link” between ape and man. The discovery was later shown to be a forgery produced by combining ape and human skulls [6]. Later, skin transplantation was fraudulently claimed by painting black ink onto a mouse [7]. Modern day science is dogged with studies where images are manipulated and journals have to devise methods to uncover such falsification [8], [9].

How common is fraud? For rather obvious reasons, it is difficult to obtain an accurate figure. A recent meta analysis has approached this question in considerable detail [10] and points out that 0.02% of papers are retracted because of fraud, a value which therefore gives a minimum estimate of the incidence of fraud. Of course, this may just be the tip of the iceberg with much more fraud either undetected or simply not retracted. The Journal of Cell Biology reported that 1% of papers submitted to it had improperly manipulated images indicating that the real figure may be greater. Another approach to obtain an estimate is to send out confidential questionnaires to scientists about fraud, remembering, of course, that not everyone will admit to it. Analysis of the questionnaires suggests that 2% of scientists admit to major research misconduct themselves and, shockingly, about 15% had seen it in others with even greater numbers engaging in “questionable practices”.

Whatever the incidence of fraud, it clearly needs addressing. It should be noted, however, that although journals and referees are now much better at spotting manipulated images and blots, this may be unlikely to stop a really determined cheat. The easiest way to commit fraud is to do a real experiment but simply lie about the drug or antibody used. That way the data is real and will survive any amount of intense scrutiny.

It is important to consider why people commit fraud. An important factor may be the pressure under which they are put to obtain results. One study, based on discussions with scientists suggested that those who worked in the most competitive universities and environments were more likely to engage in “questionable scientific practices” [11]. The desire to publish in high profile journals may also be an important factor. Indeed there is a very tight positive correlation between the Impact Factor of a journal and the fraction of papers that are retracted [12]. This correlation could have two explanations. (i) The higher the Impact Factor, the more likely the paper is to be read and therefore the more likely problems are to be noted. (ii) Alternatively, it may be that people are prepared to cut corners for the prestige of publishing in highly rated journals. These journals often seem to prefer simple, uncomplicated stories.

Pressures are put on people to publish in these, the highest rated journals. There has been recent interest in the revelation that in China cash payments are made to authors who publish in internationally rated journals [13]. These payments range from $1000 for publication in PLOS 1 to $50,000, in Nature or Science. I would ask readers of this article to think about whether their behaviour might be influenced by large sums of money. Is there a sum which would be sufficient? While it may be that the offer of $50,000 would not perturb your moral compass, would a billion be enough? It also would not be fair to focus on China. A recent letter to *Science* points out that such bonuses are seen in many countries including the USA & UK [14].

Money is not the only effective incentive. When I was a graduate student, I was delighted to have a paper accepted by main stream subject journals such as *The Journal of Physiology* and *Pflugers Archiv*. Today's students increasingly sniff at this because they have been brought up in the cult of the Impact Factor to think that only journals with double digit Impact Factors are worth entertaining. The inadequacies of the Impact Factor, an arbitrary metric which only considers citations over a two year period, as a means of estimating quality of an individual paper have been addressed sufficiently [15] that I will not labour this point here. There has been some attempt to fight this tyranny with a good example being the San Francisco Declaration on Research Assessment [16]. Nevertheless, the Impact Factor still seems to dominate. For example, there appears to be an unwritten rule in most universities in the UK that papers need to be in a journal with an Impact Factor > 5 for the work contained to be considered good enough to submit to the United Kingdom's Research Excellence Framework (REF; a mechanism for assessing the relative strength of different universities in research). We live in an age, in many universities where, not being considered for REF equates to not being considered deserving of a job. Is it therefore any great wonder that people will go a long way to publish in such journals? In some countries, departmental funding depends on a formula which explicitly takes account of the perceived quality of the journal in which a paper is published [17], [18] and it has been reported that in Germany the Impact Factor itself has been used in the calculation [19]. Worldwide, there is no doubt that a *Nature* or *Science* paper or two makes all the difference at every stage of our careers; from getting ones first faculty position all the way through promotions to the most glittering prizes. How much damage does having to retract a paper do to one's career compared to the kudos of publishing in these “top” journals? It may be that people, consciously or unconsciously, gamble and accept a certain probability of being wrong in exchange for the status of the high impact publication. As mentioned above, the fraction of papers retracted in a journal is proportional to the Impact Factor [12]. I wonder whether this is a consequence of such a gamble? Of course, many people may not set out to commit fraud. Imagine that the initial reviews of a submitted manuscript are encouraging but request a year's worth of studies. This may tempt weaker authors to include questionable data.

What can be done about the pernicious domination of the Impact Factor? I think that the answer is simple. Judge people by their scientific contributions and not by the journals in which they publish. When you describe work in a seminar or lecture, avoid giving the work extra credibility by using phrases such as “in a *Nature* paper”. The argument has been made much more eloquently by Richard Ernst who received the Nobel Prize in Chemistry for his work which underpinned NMR imaging. He said, “*And as an ultimate plea, the personal wish of the author remains to send all bibliometrics and its diligent servants to the darkest omnivoric black hole that is known in the entire universe, in order to liberate academia forever from this pestilence*” [20]. A similar point has been made by Fernandez-Delgado in an article entitled “The Index and the Moon: mortgaging scientific evaluation” which compares the Impact Factor with the Libor rate which has been used to determine interest rates, arguing that both are methodologically flawed and irreproducible [21].

A major reason that people take note of bibliometric factors such as the Impact Factor is that they are very convenient measures to use when assessing others for jobs, promotion etc. They are not a substitute for the only real test of a scientific paper; that its results stand over time and influence the work of others. Many of us work in interdisciplinary units where we have no real understanding of the quality of the work of others. It is therefore very tempting to reach for surrogates such as Impact Factors. What can be done? Again, Ernst has advice. “*And there is indeed an alternative: Very simply, start reading papers instead of merely rating them by counting citations*!” [20]. (See also similar comments from Balaban [22]).

Attempts have been made to use other bibliometric indices in place of the Impact Factor. Perhaps a positive step is to see how often a paper is cited (as opposed to the citations to the journal in which it is published). However, given that the reason that a paper is cited may be to expose fault rather than comment positively, this may not be such a great advance. The h-index has been advocated as a measure of scientific productivity and influence [23]. This involves listing an individual's publications starting with the most highly cited. One counts down the list until one reaches the h^th^ publication where the number of citations is greater than or equal to h. One issue with this metric is that it is greater for people who have had longer research careers. There have also been suggestions that the value should somehow be normalized for the number of co-authors. These and other concerns have led to the introduction of other indices. A staggering 37 of these have been compared [24] and the interested reader will also find endless information online. My view is that, although these other metrics may be more sophisticated than the crude Impact Factor, they still remain a distraction from Ernst's salutary advice of reading the paper.

The final comment I want to make about fraud is to consider the way that it is investigated. The responsibility for deciding whether fraud has occurred rests, not with the journal, but, rather, with the institution, and in some cases the funder. I find it hard to think that nobody else can see the obvious conflict of interest. The person under investigation may be someone with enormous grant income who brings great prestige to the institution. It may therefore not always be in the best interest of the institution to investigate too thoroughly. Indeed the media is full of examples where inadequate investigations have been performed [25].

Serious as it is, it does not appear likely that fraud accounts for the bulk of lack of reproducibility of science. It seems that there are two major factors. (1) Uncontrolled factors in the experiments and (2) poor design and/or statistical analysis. I will consider these in turn.

## 3 Uncontrolled factors

There are many examples here.

### 3.1 Cell lines

A major concern is that many cell lines are not what they are labelled as [26]. One problem is that many studies do not provide sufficient description of the cell line used to permit other researchers to replicate the work. Perhaps more importantly, it has been estimated that between 18 and 36% of cell lines are either contaminated or incorrectly labelled. For example, over 300 studies had used a breast adenocarcinoma cell line before it was found to be derived from human ovarian carcinoma cells. $100 million of research funding may have been spent using this misidentified cell line alone [26]. An obvious solution to such problems with cell lines is to genotype them but this is certainly not a widespread practice.

### 3.2 Use of animals

Problems with differences of strains, environment and diet may make it difficult to reproduce data. Indeed one study found that simply switching mice from soy to casein based diets had an enormous effect on hypertrophic cardiomyopathy and gene expression [27]. In this context, the use of genetically modified animals is obviously an enormously powerful technique but problems do have to be avoided. These include ensuring that the strain of the wild type is identical to that of the transgenic ones. Interpretation of the importance or otherwise of a knocked out gene may be complicated by developmental compensations in the mouse. The ARRIVE guidelines have been proposed to improve reporting of a standardized set of about 20 matters to do with the use of animals in an attempt to improve reproducibility [28]. There is, however, considerable frustration that, despite these guidelines, most papers still provide inadequate information and it has been suggested that a slimmed down version might be more readily adopted [29].

### 3.3 Chemical probes

This is a contemporary term for compounds that are inhibitors and agonists. The concern today (as has always been the case) is that these agents affect reactions other than those they are designed to target. Related to this is the phenomenon of good probes being used at too high a concentration where off target effects occur. Pharmacologists are well used to dealing with such problems. Arrowsmith et al. have pointed out that some probes are well known to be non-selective but are still used and go on to state “*...the selection of a probe compound seems to be guided by precedent and availability rather than appropriateness or quality*” [30]. Ideally, they argue, one should use at least two active probes, with different chemical structures and two, chemically related, inactive ones. They also point out that it is unfortunate that some of the better probes are not freely available to all.

## 4 Design, analysis and statistics

Many of the problems of experimental design and statistical analysis have been summarized in reports from the NIH [31] and the Academy of Medical Sciences [32]. There are many issues; a major one may be that we are victims of previous success inasmuch as we have already picked the low hanging fruit and found many of the big effects. Sophisticated modern techniques allow the study of small effects. There is a real need to do this since it is likely that many clinically important conditions result from comparatively small changes of various parameters. For example, if we are interested in the development of heart failure, we need to know the effects of infarcting 10% of the heart as opposed to 90%.

### 4.1 Comparison between animals

I wonder how much of the problem of design and analysis is due to the fact that, more and more, studies compare *between* animals: for example transgenic vs wild type; operated vs sham. In earlier times one would have made a measurement under control conditions on a tissue, repeated the measurement on the same tissue under different conditions (perhaps removing an ion or adding an inhibitor) and then often done a washout or recontrol. A classic example is the demonstration by Hodgkin and Huxley that removing Na^+^ ions reversibly abolishes the inward component of current in the squid axon [33]. Similarly, in neuroscience, researchers often record from a cell before, during and after stimulating somewhere else in the brain. If one gets a reversible change of the parameter being measured in every experiment then it is hard to see that much more is required by way of statistical analysis. The situation has now changed in many studies and a good example is provided by work on heart failure. One group of animals has heart failure induced experimentally and then tissues or cells are compared between control (sham) and heart failure animals. Another, more general, example is when tissues are compared between wild type and transgenic animals.

Examination of publications using such studies reveals questionable methods of analysis [31]. Commonly, people make measurements from several cells from each animal. They then do statistics on the population of heart failure cells compared to those from control or the population of transgenic compared to those from wild type. This means that each cell is treated as a separate experiment. In other words, the “n number” is equal to the number of cells. This cannot be valid. Imagine that one HF animal is being compared with one control but 100 cells are studied from each. Clearly n does not equal 100. Similar problems have been pointed out in other areas of basic science including neuroscience [34]. The phenomenon is called pseudoreplication and arises because technical replicates are confused with biological ones. There are more sophisticated ways of analysing the data, such as mixed linear modelling [35] and these approaches should be used. It may consequently be that more animals have to be used. At first sight this might seem to be inconsistent with the aim of reducing the number of animals used. Set against this, however, is the probability that, at present, animals are wasted, not only in the initial study, but also in subsequent work because of inappropriate statistics. A revised approach may therefore actually decrease animal usage.

What other statistical issues should one be worried about?

### 4.2 False negatives and positives

False negatives result from underpowered work which may not reveal a biologically significant effect. A typical question is whether a change of one variable results from a change of another. A good example might be why the amplitude of the systolic Ca transient changes? Imagine that the hypothesis is that the mechanism is due to a change of sarcoplasmic reticulum (SR) Ca content. The way to test this then is to measure SR Ca and see if it changes [36]. The problem comes if one finds no statistically significant change of SR content. Does this mean that a change of SR Ca is not the causal mechanism or, simply, that the measurement is not sufficiently precise? One obviously needs to know something about the system. How much of a change of SR Ca would be required to explain the result and how does this compare with the precision of the experiment? This sort of approach is obviously much more difficult to use if little is known about the system.

False positives (also called false discovery rate) are when the experiment leads to a conclusion that there is an effect whereas, in reality, none exists. Colquhoun points out that people are misled by *t*-tests [37] and often think that p < 0.05 means that there is only a 5% possibility that the result is due to chance. He argues that, particularly with small sample sizes, the chance can be much greater than that and, with typical sample sizes of under 10, p < 0.05 may make one wrong about one third of the time.

### 4.3 P hacking

This is the practice of stopping collection of data when it becomes significant. In other words, one performs a small number of experiments and persists until p < 0.05 [38]. Obviously the problem is that p will occasionally be < 0.05 even if there is no real effect and these conditions will be selected for.

### 4.4 Lack of randomisation and blinding

How do we decide which animal to use in the experimental rather than the sham group? Is there a danger of subconsciously taking more active animals for one rather than the other? Related to this, one can argue that experimenters working on tissues or cells should not know the type of animal it comes from. This is problematic if one is isolating cells from a heart from a heart failure animal when the heart may obviously be bigger. In a large enough laboratory one could have different people doing the cellular experiments than those who isolate, for example. How many of us, however, have this sort of infrastructure? Indeed, in some cases, such blinding is impossible. A good example is provided by studying the effects of pregnancy on Ca signalling or ionic currents in the myometrium. Given that the length of uterine myocytes increases 10 fold in pregnancy, the experimenter will know which type s/he is working on.

### 4.5 Publication bias

This is the problem that people tend not to report negative results. Therefore the literature will end up with an overrepresentation of positive results. This leads to an interesting consequence relating to what may happen in a field where many laboratories are working independently. At first sight one might think that this would improve the precision. Ioannidis, however, argues that it will actually *increase* the chance of getting an incorrect positive result [2]. This is because, one of the groups will get such a result by chance and this positive result will be published more easily than the negative ones and will then dominate the field. Obviously this problem could be overcome by many groups working together on the same problem. It would, however, require a major change in scientific culture.

### 4.6 HARKING

This is an acronym for “hypothesising after the facts are known” rather than before the data have been collected [39], [40]. My experience is that this is very common in cardiovascular (and other biomedical) research when the final paper and the hypothesis that it tests may bear no relationship to the original grant application or reason for doing the work. The proposed solution is to use the results to propose a hypothesis and test this with newly gathered experimental data. Obviously this will result in greater time and cost before a paper can be written. It has been argued that there should be a distinction between “exploratory, hypothesis-generating” and “confirmatory, hypothesis-testing” research [41], [42]. This is already the case in the area of clinical trials where the hypotheses must be defined before the study begins and details of the trial registered.

## 5 The role of journals

As well as authors, journals must take some responsibility for the problems mentioned above. They advertise their Impact Factors and take steps to increase them. These range from publishing more review articles (which are cited more than original papers), listing papers published in that journal in the two year period covered by the Impact Factor through to coercing authors to cite papers from the journal. It is likely that there is an unspoken Faustian Pact between authors and journals since both benefit from an increase of Impact Factor [43]. It may even be in the interest of the journal to publish erroneous, controversial work. The Impact Factor only considers the two calendar years after the publication year and, so long as any retraction occurs later, the Journal's Impact Factor will benefit from all the citations. Finally, many journals demand that the work published has novelty. This immediately makes it difficult to publish confirmatory studies. While nobody would suggest that the literature should be filled up with dozens of papers with identical results, given the problems of reproducibility reviewed above, it would seem only sensible to publish some confirmatory papers, as well as those which cannot reproduce the original finding. In this context one should applaud the approach of journals such as those from the PLoS stable which do not demand novelty.

## 6 What is the way forward?

One suggested solution is to attempt to replicate the results of published work. Ioannadis argues that “*large studies with minimal bias should be performed on research findings that are considered relatively established, to see how often they are indeed confirmed. I suspect that several established “classics” will fail the test*” [2]. In the past this would have been difficult as (see above) most journals refused to publish replication studies. Attitudes have changed and PLoS ONE has introduced a “Reproducibility Initiative” where scientists can send their work to be reproduced and, if it is, use this a hallmark of quality [44]. Nevertheless, it is probable that most scientists would still prefer to be investing their time (and grant money) in novel discovery as opposed to replication.

NIH will enforce training in experimental design. Grant reviewers are now asked to check for experimental design (randomization, blinding, validation of cell lines and antibodies etc). Reviewers are assigned the task of assessing the “*scientific premise*” of the work, i.e. how sound is the underpinning work [5]. This does not simply include pilot data but, also, previously published work. It has also been suggested that universities should be audited for scientific practice in a similar way as they are for financial matters. Others have expressed sensible caution. Noting that NIH is considering making validation compulsory, Bissell points out that validation can sometimes take a long time (and for really novel work) be very difficult [45]. In my own scientific area of electrophysiology, how would Neher and Sakmann's original single channel study [46] have been validated at a time when they were the only people who had a patch clamp?

## 7 Conclusions

It has recently been argued, by analogy with evolution, that the way that science is organized encourages bad science. The idea is that a “natural selection” for high publication rates leads to more false discoveries [47]. Modelling using optimization theory suggests that “*researchers aiming to maximize their fitness* [publication record and career success] *should spend most of their effort seeking novel results and conduct small studies that have only 10-40% statistical power. As a result, half of the studies they publish will report erroneous conclusions*” [48]. This dystopian view means that any improvement in the reproducibility situation will require a wholesale overhaul of the scientific landscape. I began by drawing a distinction between, on the one hand, fraud and, on the other, poor experimental design, execution and analysis. As I reach the end of this article, I am less and less convinced that this distinction is helpful. To persist in using statistical methods which have been shown to increase the probability of errors is to implicitly accept a higher chance that the published paper will mislead the scientific community.

So in conclusion, what is the way forward? I don't think that the option of doing nothing is a good one. I am sure that would result in funders imposing draconian conditions on us. I think that we as scientists, societies and journal editors and reviewers have to think about what reforms are needed. The exact changes may well be different in different fields. Indeed, some of us will be more affected than others.

## Disclosure

No conflict of interest.

## Funding

The author is a supported by a British Heart Foundation Chair (grant number: CH/2000004/12801).
